# Recurrent upper respiratory tract infections in early childhood: a newly defined clinical condition

**DOI:** 10.1186/s13052-024-01600-5

**Published:** 2024-02-16

**Authors:** Antonio Corsello, Gregorio Paolo Milani, Marina Picca, Roberto Buzzetti, Romeo Carrozzo, Mirko Gambino, Giovanni Chiaffoni, Paola Marchisio, Chiara Mameli

**Affiliations:** 1 Department of Clinical Sciences and Community Health, University of Milan, Milan, Italy; 2 Fondazione IRCCS Ca’ Granda Ospedale Maggiore Policlinico, Milan, Italy; 3 Italian Primary Care Paediatrics Society (SICuPP), Lombardy, Italy; 4 Clinical epidemiologist, Bergamo, Italy; 5 Department of Pediatrics, Vittore Buzzi Children’s Hospital, Milan, Italy; 6 University of Milan, Via della Commenda 9, 20122 Milan, Italy; 7 Department of Biomedical and Clinical Science “L. Sacco”, University of Milan, Milan, Italy

## Abstract

**Background:**

Recurrent Upper Respiratory Tract Infections (R-URTIs) pose a significant challenge in pediatric healthcare, affecting both children and their families. This study aimed to investigate the prevalence, risk factors, and clinical implications of R-URTI in children aged 0–5 years.

**Methods:**

This observational study involved a sample of 483 children aged 0–5 years, focusing on establishing a practical and dynamic definition of R-URTI. Family pediatricians prospectively collected socio-demographic information, medical history, and recorded the occurrence of URTI episodes. Children were followed from recruitment until March 2021, predating the COVID-19 outbreak.

**Results:**

A substantial prevalence of R-URTIs was found, estimating it at 5–10% among this age group. To define R-URTI, a practical and dynamic criterion was proposed: children experiencing a minimum of four URTI episodes, each lasting four days or more, within a six-month period, with intervals of well-being in between.

**Conclusions:**

The study highlighted that specific risk factors for R-URTI were elusive, suggesting that this condition may affect children regardless of their family or clinical history. Moreover, the study’s stratification by age group and times of observation facilitated patient-specific clinical decision-making. The proposed definition may represent a valuable tool for clinicians in diagnosing and addressing R-URTI cases.

**Supplementary Information:**

The online version contains supplementary material available at 10.1186/s13052-024-01600-5.

## Introduction

Recurrent respiratory tract infections (RRTIs) represent a widespread childhood medical condition that poses significant challenges to pediatric healthcare providers. These infections affect a substantial proportion of infants and children aged 1–6 years, accounting for up to 25% of cases [1, 2]. During early childhood, RRTIs often trigger medical consultations and emergency room visits, significantly impacting the well-being of affected children and their families, and frequently resulting in school absences [3].

Moreover, RRTIs impose a substantial burden on public health, necessitating recurrent medical assessments, frequent antibiotic treatments and, in some cases, hospitalizations [1, 4, 5]. Several risk factors may contribute to the development of RRTIs, including early exposure to infectious agents, immune system immaturity, indoor and outdoor pollution, secondhand smoke exposure, atopy and allergy, and low socio-economic status [1, 6].

Despite the clinical significance of RRTIs, the scientific community lacks consensus on its definition [3, 5, 7, 8]. Moreover, some children exclusively experience recurrent upper respiratory tract infections (R-URTIs), but the number of infections required to classify them as recurrent remains unknown. The differentiation between RRTIs and R-URTIs could be speculated considering that that both the risk factors associated with these two conditions and their clinical burden may differ [9–12]. Therefore, we postulated that the incidence and specific risk factors of R-URTI may warrant its classification as a distinct clinical entity.

For this purpose, we initiated a prospective cohort study in a primary care setting. Our main objectives were to estimate the actual prevalence of URTIs in preschool children aged 0–5 years. Subsequently, we aimed to identify the actual prevalence and potential risk factors for R-URTI events in children without underlying predisposing conditions. Finally, we sought to propose a practical definition for R-URTI based on these findings.

## Materials and methods

This prospective, observational cohort study was performed between October 2019 and February 2020 in a primary care setting in Lombardy, Italy.

### Study population

This study was performed involving a convenience sample of 69 family pediatricians working for national public health system and providing free medical care, and who were member of the Italian Primary Care Pediatrics Society (SICuPP), Lombardy section, joined the study as volunteers. Children aged 0–5 years cared by involved family pediatricians were considered eligible for the study. Children with prematurity (gestational age < 37 weeks), congenital abnormalities of the respiratory tract, congenital or acquired immunodeficiency, cystic fibrosis, cardiovascular, renal or hematological diseases were excluded. Medical history was collected using a specific questionnaire during the first medical visit, and the exclusion of comorbidities aimed at ensuring a homogenous study population, in order to investigate the impact of respiratory infections in an otherwise healthy cohort, minimizing confounding factors related to other diseases.

### Study protocol

At recruitment, the family pediatrician completed a case report form (CRF) for each enrolled child which included socio-demographic information about each child. Furthermore, information on the gestational age, type of childbirth and early feeding (breastfeeding, formula, mixed feeding), number and type of vaccinations, exposure to passive smoking, number of siblings, parental age, attendance in the community, active or familiar history of allergies were collected. Further information on data collection is reported in the Supplementary Material.

Parents of enrolled children were instructed to contact the pediatrician in case of fever (defined as an axillary temperature > 38 °C) or flu-like symptoms [13]. A full clinical examination was then conducted and, if an URTI was diagnosed, another CRF was filled with the specific diagnosis, as previously described in other studies conducted on an analogue cohort [14, 15]. Based on the site infection, rhinitis, rhinosinusitis, pharyngotonsillitis, acute otitis media and laryngitis were considered as potential diagnosis of URTI [16–18]. Each child was followed from the day of recruitment up to March 30, 2021. Due to the occurrence of the COVID-19 outbreak, only months from October 2019 to February 2020 (corresponding to autumn and winter months) could be used for this purpose. In March 2020 Lombardy experienced one of the first and deadliest COVID-19 outbreaks in the world, forcing Health Authorities to a national lockdown [19].

### Statistical analysis

The recruited population was divided into 3 groups: children aged < 1 year, 1–2 and 3–5 years. Incidence rates of URTIs were computed for each age group to estimate the expected likelihood of experiencing a URTI episode in any given week. For each child, if symptoms were present for four or more days out of seven in a week, that week was deemed “affected by URTI.” The overall incidence rate of URTI for each age group was calculated as the total number of URTI episodes divided by the total number of weeks of observation per child. This calculation considered the possibility that some subjects transitioned between age groups during the observation period, thus contributing to the incidence rate for both age categories. Subsequently, for each child, we estimated the probability of experiencing a URTI in any given week using the binomial distribution. A R-URTI was defined as a subject who had a number of URTI episodes for which the cumulative probability of that number (or a higher number) of URTIs was less than 10% (*p* = 0.10). The probability of URTIs occurring at least “*x = k*” times in *n* weeks was determined using the formula: “P(URTI ≥ x = k) = 1 - Σ[n!/(x!(n-x)!) * p^x * (1–p)^(n-x)] < 0.10” (p = probability of URTI in one week, x = number of weeks with URTIs, k = threshold number for R-URTI, n = number of weeks of exposure).

The association between each independent variable and the development of an R-URTI was assessed. Associations were evaluated using the Chi-square test or Fisher’s exact test when expected frequencies were low (*p* = 0.05).

## Results

A total of 483 children participated in our study. The frequency of URTIs, taking into account both the number of cases and the cumulative weeks of observation across all children in various age groups, was as follows: (1) children aged < 1 year: 133 cases/1658 weeks, with a mean of 8.02 cases per 100 weeks per person; (2) children aged 1–2 years: 282 cases/3025 weeks, with a mean of 9.32 cases per 100 weeks per person; (3) children aged 3–5 years: 191 cases/2750 weeks, with a mean of 6.95 cases per 100 weeks per person.

The calculated incidence rates were 8.02%, 9.32%, and 6.95% for the < 1 year, 1–2 years, and 3–5 years age groups, respectively. Table 1 provides a summary of the minimum number of URTIs required to classify a child as affected by R-URTIs, taking into account different observation periods between the first and the last episode and specific for the different age groups.

When considering the entire population, 44 out of 483 children (9.1%) met the criteria for a R-URTI. Among infants (age < 1 year), there were 122 participants, of whom 12 developed R-URTI (9.84%). In the 1–2 years age group, which had 219 participants, 14 children were affected by R-URTI (6.39%). In the 3–5 years age group, consisting of 186 children, 18 presented with R-URTI (9.68%). These percentages account for the fact that 44 children transitioned from one age group to the next during the observation period.

**Table 1:** Minimum events required to define R-URTI, categorized by observational period and age

N. of weeks of observation	< 1 years	1–2 years	3–5 years
3–5	2	2	2
6	2	3	2
7	3	3	2
8–12	3	3	3
13–14	3	4	3
15–16	4	4	3
17–19	4	4	4
20–21	4	5	4

Moreover, when considering possible significant risk factors associated with the occurrence of R-URTIs, community placement was found to be more related in children aged 1–2 years old (OR 14.45, *p* = 0.005); in cases of community placement since more than three months, this association was still significant (OR 7.58 *p* = 0.023), as well as subgroups including the size of the class (more than 10 children per class, OR 10.84, *p* = 0.019) and having meals at school (OR 8.55, *p* = 0.033). These factors were not confirmed to be significant in children aged > 2 years old.

Moreover, our data showed that patients aged 1–2 years old with inhalant allergy present an increased risk of developing RRTIs (OR 16.25, *p* = 0.023). No statistically significant association either between the development of R-URTIs and family history of allergies was found in any of the three groups. Type of feeding, gestational age at birth, number of siblings, parental age, vaccinations and passive smoke were not found to be associated with an increased or reduced risk of R-URTI in any group. Associations with specific risk factors for each age group are reported in the Supplementary Material.

## Discussion

This study identified a subset of children who manifest recurrent upper respiratory infections without involving the lower airways and, for the first time, attempts to provide a new clinical and epidemiology definition of R-URTI.

In 2021, an Italian intersociety consensus proposed the following classification of RRTIs after a review of scientific literature: (1) 1–3 years, 6 or more respiratory tract infections in a year, of which 1 has to be severe pneumonia or 2 mild cases of pneumonia confirmed by clinical or radiological criteria; (2) 3–6 years, 5 or more respiratory tract infections in a year, including at least either 1 episode of severe pneumonia or 2 mild ones (confirmed by clinical or radiological criteria); (3) 6–12 years, 3 or more respiratory tract infections (including either 1 severe or 2 mild pneumoniae, as previously stated) [10]. Children with chronic diseases such as cystic fibrosis, primary ciliary dyskinesia, bronchiectasis, cardiorespiratory malformations, or neuromuscular disorders are excluded from this definition. Specific definitions have also been provided for particular respiratory diseases. For instance, acute otitis media is considered recurrent if it occurs more than three times in six months (or four times in one year) [20]. Although classifications are relevant for clinical practice, they may not characterize those children who present several episodes of URTIs but do not fit any specific existing definition. Furthermore, it is important to point out that some of the studies included to join this definition of RRTIs were conducted even decades ago, when circulation of infectious agents, available preventive measures and environmental exposure were significantly different [10].

Our findings also provide an estimate of the R-URTI in children aged 0–5 years old that rages from 5 to 10%. This discovery represents a pivotal aspect of our research, highlighting the substantial burden of upper RRTIs in young children. This study did not identify specific risk factors for R-URTI. This finding has at least two implications: (1) it suggests that R-URTI might affect potentially all children regardless of their family or clinical history; (2) the frequency of URTI events may be the most crucial criterion to consider in definition of R-URTI.

Assessing the frequency of URTIs may be a challenge for the clinician. Indeed, the observation and duration of URTI events can be highly heterogeneous, influenced by factors such as parental reporting accuracy, the unpredictable nature of URTIs, and the diverse manifestations of these infections. Moreover, the considerable variability in URTI episodes further complicates the task of assessing frequency.

To address these challenges, our study introduced a practical and dynamic tool for categorizing patients between the ages of one month and five years as having upper RRTIs. The dynamic observation period reported in our thresholds, ranging from 3 to 21 weeks, may allow pediatricians to early identify cases of R-URTI. Overall, we suggest the diagnosis of R-URTI in otherwise well-being children up to 5 years of age experiencing at least 4 URTI episodes lasting four days or more within a six-month period. The episodes must be separated from each other by a period of well-being. New studies should test the validity of this new definition.

Our study has inherent limitations, notably the narrow time frame from October 2019 to February 2020 for data collection. Unfortunately, the COVID-19 pandemic’s unprecedented outbreak in Lombardy, Italy, led to nationwide lockdowns, school closures, and disruptions to healthcare services, rendering data collection beyond February 2020 unfeasible [21, 22]. Consequently, our study did not account for lower respiratory infections, which would have provided a more comprehensive understanding of RRTIs in children, as well as other possible risk factors such as the mode of delivery. Moreover, we have not included in our data possible ENT evaluations or anatomical anomalies that may predispose to the development of URTIs.

On the other hand, the stratification of results by age group not only enriches data interpretation but also facilitates age-specific clinical decision-making. This stratification provides clinicians with an additional tool to diagnose the single patient with R-URTI based on the number of weeks between the first and last episode of URTI during the first 5 years of life, regardless of the duration of the observation period.

In this study, we emphasized the importance of considering the frequency of URTI events as a central criterion for defining and addressing R-URTI in clinical practice. This differentiation may offer a specific understanding of pediatric RRTIs, advocating for a more nuanced approach to their management and prevention.

## Conclusions

In this study, we observed a significant prevalence of R-URTI in children aged 0–5 years, with estimates ranging from 5 to 10%. To address the lack of a consensus on its definition, we proposed a practical and dynamic definition of R-URTI, suggesting that children within this age group experiencing a minimum of four episodes within a six-month period could be classified as having R-URTI. This clinical entity not only underscores the substantial burden of URTI in young children but also offers a specific criterion for clinicians to identify and manage cases of R-URTI, facilitating a more nuanced approach to its early diagnosis. Further research is needed to validate this proposed definition and enhance our understanding of recurrent infections in children without specific risk factors.

### Electronic supplementary material

Below is the link to the electronic supplementary material.

Supplementary Material 1

## Data Availability

Data and consents are available from the corresponding author upon reasonable request.

## Abbreviations

R-URTI: Recurrent upper respiratory tract infection
RRTI: Recurrent respiratory tract infection
SICuPP: Italian Primary Care Paediatrics Society
