# Supplementary material for: Recurrent upper respiratory tract infections in early childhood: a newly defined clinical condition
Source: Ital J Pediatr. 2024 Feb 16;50:30. doi: 10.1186/s13052-024-01600-5 (PMC10873936; doi:10.1186/s13052-024-01600-5)
Supplement: Supplementary file 1 — Supplementary Material 1 [file 13052_2024_1600_MOESM1_ESM.docx]

**Supplementary Table 1.** Risk factors for R-URTI, age group < 1 year.

| Age group: < 1 year  (122 subjects, 12 R-URTI) | R-URTI | | No R-URTI | | OR | 95% C.I. | | p value |
| --- | --- | --- | --- | --- | --- | --- | --- | --- |
|  | **n** | **exposed** | **n** | **exposed** |  | **lower** | **upper** |  |
| Age ≥ 36. mother | 12 | 7 | 105 | 52 | 1.43 | 0.41 | 4.61 | 0.393 |
| Age ≥ 36. father | 12 | 2 | 105 | 43 | 0.29 | 0.07 | 1.53 | 0.089 |
| Family history of inhalant allergy | 12 | 5 | 107 | 39 | 1.25 | 0.36 | 4.10 | 0.475 |
| Family history of food allergy | 12 | 0 | 107 | 5 | 0.75 | 0.04 | 14.71 | 0.582 |
| Family history of other types of allergies | 12 | 1 | 106 | 3 | 3.12 | 0.25 | 27.09 | 0.353 |
| Having siblings | 12 | 8 | 105 | 56 | 1.75 | 0.47 | 5.84 | 0.286 |
| N. of rooms (≥ 4) in the house | 12 | 1 | 103 | 30 | 0.22 | 0.03 | 1.99 | 0.112 |
| N. of people (≥ 3) living in the house | 12 | 9 | 104 | 75 | 1.16 | 0.29 | 4.52 | 0.567 |
| Smoke, mother | 12 | 12 | 104 | 94 | 2.78 | 0.14 | 46.61 | 0.320 |
| Smoke, father | 12 | 10 | 102 | 74 | 1.89 | 0.37 | 8.66 | 0.339 |
| Sex, male | 12 | 8 | 109 | 54 | 2.04 | 0.54 | 6.69 | 0.206 |
| Gestational age ≥ 37 weeks | 12 | 12 | 107 | 102 | 1.34 | 0.07 | 25.02 | 0.582 |
| Formula feeding | 12 | 0 | 106 | 12 | 0.30 | 0.02 | 5.91 | 0.258 |
| Mixed feeding | 12 | 6 | 94 | 48 | 0.96 | 0.29 | 3.20 | 0.593 |
| Exclusive breastfeeding, duration (≥ 6 months) | 3 | 3 | 42 | 30 | 2.87 | 0.13 | 55.70 | 0.384 |
| Allergy, inhalants | 12 | 0 | 106 | 0 | - | - | - | - |
| Allergy, food | 12 | 0 | 106 | 1 | 2.81 | 0.09 | 61.10 | 0.898 |
| Vaccination, hexavalent (DTaP-Hib-IPV-HepB) | 11 | 11 | 97 | 94 | 0.85 | 0.04 | 17.89 | 0.722 |
| Vaccination, MMR (Measles, Mumps, Rubella) | 11 | 0 | 97 | 20 | 0.16 | 0.01 | 3.26 | 0.093 |
| Vaccine, chickenpox | 11 | 0 | 97 | 17 | 0.20 | 0.01 | 3.95 | 0.137 |
| Vaccine, pneumococcal | 11 | 11 | 97 | 79 | 5.35 | 0.27 | 85.23 | 0.121 |
| Vaccine, rotavirus | 11 | 10 | 97 | 79 | 2.28 | 0.26 | 17.76 | 0.386 |
| Vaccine, seasonal flu | 12 | 3 | 101 | 7 | 4.48 | 0.75 | 15.63 | 0.072 |
| Community, attending | 12 | 6 | 104 | 33 | 2.15 | 0.59 | 6.59 | 0.941 |
| Community, duration of placement (≥ 3 months) | 12 | 6 | 97 | 20 | 3.85 | 0.93 | 10.96 | 0.993 |
| N. of children (≥ 10) in each class | 9 | 5 | 79 | 24 | 2.86 | 0.63 | 10.31 | 0.127 |
| Meal in community | 9 | 5 | 85 | 34 | 1.88 | 0.44 | 7.04 | 0.290 |

**Supplementary Table 2.** Risk factors for R-URTI, age group 1-2 years.

| Age group: 1-2 years  (219 subjects, 14 R-URTI) | R-URTI | | No R-URTI | | OR | 95% C.I. | | p value |
| --- | --- | --- | --- | --- | --- | --- | --- | --- |
|  | **n** | **exposed** | **n** | **exposed** |  | **lower** | **upper** |  |
| Age ≥ 36. mother | 14 | 8 | 194 | 113 | 0.96 | 0.32 | 2.87 | 0.573 |
| Age ≥ 36. father | 14 | 9 | 193 | 98 | 1.74 | 0.54 | 5.20 | 0.243 |
| Family history of inhalant allergy | 14 | 4 | 198 | 71 | 0.72 | 0.22 | 2.41 | 0.406 |
| Family history of food allergy | 14 | 0 | 195 | 9 | 0.68 | 0.04 | 12.52 | 0.529 |
| Family history of other types of allergies | 14 | 2 | 194 | 8 | 3.88 | 0.63 | 17.28 | 0.139 |
| Having siblings | 14 | 7 | 198 | 97 | 1.04 | 0.35 | 3.07 | 0.637 |
| N. of rooms (≥ 4) in the house | 14 | 5 | 195 | 60 | 1.25 | 0.40 | 3.83 | 0.452 |
| N. of people (≥ 3) living in the house | 14 | 10 | 194 | 133 | 1.15 | 0.34 | 3.77 | 0.543 |
| Smoke, mother | 14 | 12 | 199 | 172 | 0.94 | 0.20 | 4.46 | 0.595 |
| Smoke, father | 13 | 7 | 197 | 145 | 0.42 | 0.14 | 1.39 | 0.113 |
| Sex, male | 14 | 8 | 205 | 105 | 1.27 | 0.42 | 3.73 | 0.441 |
| Gestational age ≥ 37 weeks | 13 | 13 | 199 | 190 | 1.35 | 0.07 | 23.98 | 0.559 |
| Formula feeding | 11 | 3 | 197 | 37 | 1.62 | 0.40 | 6.22 | 0.356 |
| Mixed feeding | 8 | 3 | 160 | 74 | 0.70 | 0.16 | 3.07 | 0.456 |
| Exclusive breastfeeding, duration (≥ 6 months) | 8 | 5 | 115 | 77 | 0.82 | 0.19 | 3.67 | 0.535 |
| Allergy, inhalants | 14 | 2 | 197 | 2 | 16.25 | 1.12 | 66.65 | **0.023** |
| Allergy, food | 14 | 0 | 197 | 6 | 1.02 | 0.05 | 18.92 | 0.659 |
| Vaccination, hexavalent (DTaP-Hib-IPV-HepB) | 14 | 14 | 187 | 180 | 1.20 | 0.06 | 21.89 | 0.598 |
| Vaccination, MMR (Measles, Mumps, Rubella) | 14 | 11 | 190 | 136 | 1.46 | 0.38 | 5.30 | 0.415 |
| Vaccine, chickenpox | 14 | 9 | 188 | 116 | 1.12 | 0.36 | 3.44 | 0.545 |
| Vaccine, pneumococcal | 14 | 12 | 187 | 174 | 0.45 | 0.10 | 2.40 | 0.281 |
| Vaccine, rotavirus | 14 | 8 | 187 | 121 | 0.73 | 0.25 | 2.24 | 0.382 |
| Vaccine, seasonal flu | 14 | 4 | 195 | 32 | 2.04 | 0.57 | 6.51 | 0.205 |
| Community, attending | 14 | 14 | 199 | 133 | 14.45 | 0.77 | 223.48 | **0.005** |
| Community, duration of placement (≥ 3 months) | 11 | 10 | 167 | 95 | 7.58 | 0.87 | 55.56 | **0.023** |
| N. of children (≥ 10) in each class | 10 | 10 | 162 | 107 | 10.84 | 0.57 | 173.25 | **0.019** |
| Meal in community | 13 | 13 | 184 | 140 | 8.55 | 0.46 | 135.41 | **0.033** |

**Supplementary Table 3.** Risk factors for R-URTI, age group 3-5 years.

| Age group: 3-5 years  (186 subjects, 18 R-URTI) | R-URTI | | No R-URTI | | OR | 95% C.I. | | p value |
| --- | --- | --- | --- | --- | --- | --- | --- | --- |
|  | **n** | **exposed** | **n** | **exposed** |  | **lower** | **upper** |  |
| Age ≥ 36. mother | 17 | 12 | 164 | 122 | 0.83 | 0.28 | 2.53 | 0.465 |
| Age ≥ 36. father | 17 | 10 | 164 | 115 | 0.61 | 0.23 | 1.78 | 0.243 |
| Family history of inhalant allergy | 18 | 6 | 165 | 52 | 1.09 | 0.38 | 3.03 | 0.533 |
| Family history of food allergy | 18 | 0 | 165 | 8 | 0.50 | 0.03 | 9.53 | 0.429 |
| Family history of other types of allergy | 18 | 0 | 165 | 7 | 0.57 | 0.03 | 10.90 | 0.478 |
| Having siblings | 17 | 7 | 163 | 97 | 0.48 | 0.19 | 1.41 | 0.116 |
| N. of rooms (≥ 4) in the house | 17 | 6 | 163 | 62 | 0.89 | 0.32 | 2.55 | 0.523 |
| N. of people (≥ 3) living in the house | 17 | 11 | 162 | 133 | 0.40 | 0.15 | 1.30 | 0.086 |
| Smoke, mother | 17 | 17 | 166 | 146 | 4.90 | 0.26 | 77.40 | 0.127 |
| Smoke, father | 17 | 14 | 165 | 128 | 1.35 | 0.36 | 4.82 | 0.462 |
| Sex, male | 18 | 7 | 168 | 88 | 0.58 | 0.23 | 1.65 | 0.201 |
| Gestational age ≥ 37 weeks | 18 | 18 | 163 | 154 | 2.28 | 0.12 | 38.27 | 0.381 |
| Formula feeding | 17 | 3 | 160 | 34 | 0.79 | 0.22 | 2.98 | 0.507 |
| Mixed feeding | 14 | 9 | 126 | 58 | 2.11 | 0.62 | 6.18 | 0.155 |
| Exclusive breastfeeding, duration (≥ 6 months) | 7 | 4 | 97 | 63 | 0.72 | 0.16 | 3.48 | 0.482 |
| Allergy, inhalants | 18 | 1 | 165 | 4 | 2.37 | 0.22 | 19.82 | 0.408 |
| Allergy, food | 18 | 0 | 165 | 3 | 1.25 | 0.06 | 24.61 | 0.732 |
| Vaccination, hexavalent (DTaP-Hib-IPV-HepB) | 18 | 18 | 161 | 161 | - | - | - | - |
| Vaccination, MMR (Measles, Mumps, Rubella) | 18 | 18 | 161 | 154 | 1.80 | 0.09 | 31.19 | 0.470 |
| Vaccine, chickenpox | 18 | 11 | 161 | 71 | 1.99 | 0.69 | 5.04 | 0.131 |
| Vaccine, pneumococcal | 18 | 18 | 161 | 152 | 2.30 | 0.12 | 38.72 | 0.376 |
| Vaccine, rotavirus | 18 | 1 | 161 | 23 | 0.35 | 0.05 | 2.99 | 0.268 |
| Vaccine, seasonal flu | 18 | 1 | 165 | 34 | 0.23 | 0.03 | 1.94 | 0.103 |
| Community, attending | 17 | 15 | 166 | 150 | 0.80 | 0.17 | 3.90 | 0.518 |
| Community, duration of placement (≥ 3 months) | 13 | 10 | 112 | 94 | 0.64 | 0.17 | 2.69 | 0.377 |
| N. of children (≥ 10) in each class | 15 | 14 | 146 | 144 | 0.19 | 0.02 | 3.12 | 0.256 |
| Meal in community | 18 | 17 | 165 | 162 | 0.31 | 0.04 | 3.86 | 0.341 |
